# ‘I just didn’t find time to exercise’: Co-designed physical activity resources for young Australian shiftworkers

**DOI:** 10.1093/heapro/daaf175

**Published:** 2025-11-04

**Authors:** Madeline Sprajcer, Alexandra E Shriane, Sally A Ferguson, Charlotte C Gupta, Ruby G Smith, Jeannie J Kim, Crystal L Baum, Tracy Kolbe-Alexander, Robert Stanton, Matthew J W Thomas, Jessica L Paterson, Chloe Gallagher, Gabrielle Rigney, Grace E Vincent

**Affiliations:** Appleton Institute and School of Health, Medical and Applied Sciences, Central Queensland University, 44 Greenhill Road, Wayville, SA 5034, Australia; Appleton Institute and School of Health, Medical and Applied Sciences, Central Queensland University, 44 Greenhill Road, Wayville, SA 5034, Australia; Appleton Institute and School of Health, Medical and Applied Sciences, Central Queensland University, 44 Greenhill Road, Wayville, SA 5034, Australia; Appleton Institute and School of Health, Medical and Applied Sciences, Central Queensland University, 44 Greenhill Road, Wayville, SA 5034, Australia; Appleton Institute and School of Health, Medical and Applied Sciences, Central Queensland University, 44 Greenhill Road, Wayville, SA 5034, Australia; Appleton Institute and School of Health, Medical and Applied Sciences, Central Queensland University, 44 Greenhill Road, Wayville, SA 5034, Australia; Appleton Institute and School of Health, Medical and Applied Sciences, Central Queensland University, 44 Greenhill Road, Wayville, SA 5034, Australia; School of Health and Medical Sciences and Centre for Health Research University of Southern Queensland, PO BOX 4393 Raceview 4305, Ipswich, QLD, Australia; Manna Institute, Centre for Health Research, University of Southern Queensland, PO BOX 4393 Raceview 4305, Ipswich, QLD, Australia; Department of Human Biology, Division of Exercise Science and Sports Medicine, Faculty of Health Sciences, University of Cape Town, Boundary Road, Newlands, Cape Town, 7725, South Africa; Appleton Institute and School of Health, Medical and Applied Sciences, Central Queensland University, 44 Greenhill Road, Wayville, SA 5034, Australia; Cluster for Resilience and Wellbeing, Appleton Institute, Central Queensland University, 44 Greenhill Road, Wayville, SA 5034, Australia; Appleton Institute and School of Health, Medical and Applied Sciences, Central Queensland University, 44 Greenhill Road, Wayville, SA 5034, Australia; Flinders Institute for Mental Health and Wellbeing, Flinders University, Sturt Road, Bedford Park, SA 5042, Australia; Research Institute for Sport and Exercise Sciences, Liverpool John Moores University, Tom Reilly Building, Byrom Street Campus, Liverpool L3 3AF, United Kingdom; Appleton Institute and School of Health, Medical and Applied Sciences, Central Queensland University, 44 Greenhill Road, Wayville, SA 5034, Australia; Appleton Institute and School of Health, Medical and Applied Sciences, Central Queensland University, 44 Greenhill Road, Wayville, SA 5034, Australia

**Keywords:** co-design, health promotion, workplace health, participatory research, behaviour change

## Abstract

Shiftworkers are vital to essential industries yet often experience adverse health impacts, including barriers to regular physical activity. Young shiftworkers face additional challenges due to the transitional life stage and the increase of unhealthy behaviours. This study aimed to co-design tailored, evidence-based physical activity resources to support the health and wellbeing of young shiftworkers. A participatory co-design approach was undertaken, involving 48 co-designers, including young, experienced, and former shiftworkers, workplace health and safety professionals, science communicators, and academic experts. Participants attended 1–2 of eight online workshops. Data from the recorded and transcribed workshops informed resource development and included the identification of key physical activity topics and effective communication strategies. A combined inductive and deductive thematic analysis identified 22 unique codes, which were synthesized into five major themes: physical activity basics, impacts of insufficient physical activity, physical activity for shiftworkers, strategies and actions for shiftworkers, and recommendations for workplaces. These themes guided the development of a public-facing website containing evidence-based, context-relevant physical activity resources tailored specifically for young shiftworkers. A motivational and positive framing was consistently endorsed to enhance engagement and support behaviour change. The final resources reflect the diverse perspectives of co-designers and offer a scalable and practical tool to promote physical activity in this priority population. Further research should investigate resource uptake, usability, and behavioural outcomes over time.

Contribution to Health Promotion

- Co-designed physical activity resources were created with young shiftworkers to reflect their lived experiences and needs.
- A strengths-based, motivational approach was recommended to increase engagement and avoid fear-based messaging.
- Resources highlight practical, evidence-based strategies for fitting activity into complex and irregular shiftwork schedules.
- The free online resources (www.healthyshiftwork.com) aim to promote increased physical activity for young shiftworkers worldwide.
- Findings support a whole-of-system approach, encouraging workplaces to create environments that make physical activity easier and more accessible.

## INTRODUCTION

Shiftwork, defined as employment outside standard daytime hours (e.g. outside 9:00 a.m. to 5:00 p.m.), can include early morning, evening, and night shifts (Atkinson *et al*. 2008). Globally, an estimated 15%–30% of workers engage in shiftwork, including in Australia (Australian Bureau of Statistics 2024), the European Union (Eurofound 2017), and the United States of America (Bureau of Labor Statistics 2019). Industries such as healthcare, transportation, utilities, and manufacturing depend heavily on shiftworkers to maintain production and/or essential 24-hour services (Rajaratnam and Arendt 2001, Folkard and Tucker 2003). However, this reliance can come at a cost, as shiftworkers face significantly higher risks of adverse health and wellbeing outcomes compared to daytime workers (Souza *et al*. 2019). Studies have consistently linked shiftwork to poorer physical and psychological health, an increased risk of chronic diseases, and premature mortality (Nea *et al*. 2015, Kecklund and Axelsson 2016, Torquati *et al*. 2018, 2019, Silva *et al*. 2020, Crowther *et al*. 2021). Given these outcomes, equipping shiftworkers with practical strategies for optimizing their health and reducing these risks is crucial. One such approach is improving the amount of physical activity undertaken.

Physical activity is critical for maintaining physical and mental health (Rhodes *et al*. 2017, Mahindru *et al*. 2023). Regular physical activity can reduce the risk of musculoskeletal injuries, in addition to sprains and strains, which are common in shiftworkers (Stimpfel *et al*. 2015, Flahr *et al*. 2018, Härmä *et al*. 2020). Sleep quality is also generally improved by undertaking physical activity (Kline 2014). Shiftworkers are more likely than day workers to experience both insufficient physical activity (i.e. not undertaking enough physical activity, particularly during leisure time) and too much sedentary time (i.e. time spent sitting, regardless of the amount of overall physical activity undertaken) (Loprinzi 2015). Insufficient physical activity and sedentary behaviour are key risk factors for chronic conditions such as cardiovascular disease, diabetes, obesity, and poor mental health, concerns particularly relevant to shiftworkers (Lowden *et al*. 2010, Loprinzi 2015, Wahid *et al*. 2016, Elagizi *et al*. 2020, Mahindru *et al*. 2023). Meeting the World Health Organization’s recommendation of 150 min of moderate-intensity physical activity per week is essential for preventing non-communicable diseases (World Health Organization 2010).

For shiftworkers, the risk of developing a chronic and/or serious health condition is impacted by a range of factors. Access to exercise facilities such as gyms and pools is often limited, particularly for those in areas where facilities with extended opening hours are unavailable (e.g. rural or remote shiftworkers), and when shift schedules, facility opening hours, or the timing of specific services (e.g. exercise classes) are misaligned (Sweeney *et al*. 2025). Additionally, irregular sleep and circadian disruption associated with shiftwork frequently lead to fatigue and decreased motivation for physical activity (Foster 2020). Moreover, irregular schedules can make it challenging to establish a regular exercise routine, as can the addition of family and other personal responsibilities on top of non-standard hours of work (Cheng *et al*. 2020). Although some shiftworkers meet recommended physical activity levels through occupational tasks, their irregular work schedules often prevent them from obtaining sufficient sleep and recovery (Chappel *et al*. 2017). Together, these competing demands create significant barriers to maintaining sufficient physical activity.

Young adult shiftworkers (i.e. those aged 18–25 years) have been identified as a population with higher risk of reduced physical activity behaviours due to the demands of shiftwork (Crowther *et al*. 2024). This is particularly concerning, given this age group represents the highest proportion of shiftworkers in Australia, with one in five shiftworkers aged under 25 years (Australian Bureau of Statistics 2023). Early adulthood is a period often associated with an increase in unhealthy behaviours (e.g. poor eating habits, insufficient physical activity), particularly as young adults transition into full-time employment (Kwan *et al*. 2012, Winpenny *et al*. 2020). This transition can be influenced by new daily routines and responsibilities, in addition to increased autonomy and access to resources such as disposable income, which can lead to increased engagement in risky behaviours, including substance misuse and decreased participation in health-promoting activities such as regular exercise (Corder *et al*. 2019). The consequences of low physical activity levels are exacerbated by increased sedentary behaviour, poor sleep quality, and poor mental health outcomes observed shortly after shiftwork commencement (Betson *et al*. 2022, Crowther *et al*. 2024, Harris *et al*. 2024). Developing and implementing tailored physical activity strategies and advice specific to the unique challenges faced by young shiftworkers (YSW) is therefore essential for supporting their long-term health and wellbeing.

Interventions, including those that are educational in nature, are sparse in the context of physical activity for shiftworkers (Dos Reis *et al*. 2025). A recent systematic review identified only six publications describing existing physical activity interventions, all of which used different intervention styles and approaches (e.g. educational resources, coaching, text-messaging, one-off in-person training)—and all of which appear to show potential to improve physical activity outcomes (Dos Reis *et al*. 2025). Beyond physical activity, health interventions developed for shiftworkers that incorporate educational components have demonstrated effectiveness in reducing negative health outcomes (Booker *et al*. 2022). For example, sleep education has resulted in significant increases in sleep duration (Crowther *et al*. 2021), in addition to reduced symptoms of insomnia, anxiety, and depression in shiftwork populations (Booker *et al*. 2022). However, the majority of these existing interventions focus on sleep rather than physical activity (Neil-Sztramko *et al*. 2014, Robbins *et al*. 2021). As such, there is a clear need for novel, tailored educational resources targeting physical activity in shiftworkers.

Evidence shows that for physical activity interventions to be effective in shiftworkers, shiftworkers themselves should be involved in the development of the intervention (Demou *et al*. 2018, Dos Reis *et al*. 2025). Evidence from these studies suggests that it is essential to consider factors influencing uptake and engagement when developing tailored physical activity resources. Tailoring and user engagement may be even more critical for YSW in particular, as young people exhibit greater engagement with physical activity interventions when features such as customization and personalization are included (Schwarz *et al*. 2023). Research also indicates that in general, shiftworkers have limited interaction with generic health materials but are more likely to engage with content specifically tailored to their unique lifestyles (Caruso 2014, Thorne *et al*. 2025). Given the diversity of approaches trialled to date and the inconsistency in intervention delivery methods, there is a need to first understand what types of content and communication strategies are most relevant and engaging for YSW. This foundational step will inform the design of future, more comprehensive interventions that incorporate these strategies effectively. To ensure that the physical activity resources developed as part of the present study are both relevant and effective, a participatory co-design methodology was employed to explore the following research question:

What physical activity content and communication strategies do a group of co-designers (young, experienced, and previous shiftworkers (PSW), workplace health and safety experts, and science communications (SC) specialists) consider to be important and relevant for YSW?

## MATERIALS AND METHODS

The present study used a participatory, co-design methodology to develop a suite of resources for YSW addressing their sleep, physical activity, and nutrition needs. This approach involved multiple rounds of workshops with shiftworkers (young, experienced, and previous) and relevant experts [e.g. work health and safety (WHS) experts, science communication (SC) experts, academic experts (AE)], allowing for iterative feedback, power-sharing, and collaborative decision-making. Co-designers were actively involved in shaping the content, format, and delivery methods used within the resources, ensuring they were contextually relevant, accessible, and aligned with the needs of YSW. The process was iterative, beginning with workshops to identify key content areas, followed by additional sessions to refine prototype materials and draft versions of the resources. This co-design process was guided by principles of inclusivity, reflexivity, and shared ownership.

All findings related to the development of sleep (Shriane *et al*. 2025) and nutrition (under review) resources are reported separately, whilst an exploration and evaluation of the co-design methodology used is also published elsewhere (Shriane *et al*. 2024). The broader study was separated into these component publications to ensure that each could be described and analysed in sufficient detail to provide a meaningful contribution to the evidence-base. While each of the three topics were discussed and content generated via the same workshop process, and analysed simultaneously, no content related to findings is duplicated across publications. The co-design evaluation found that the co-designers viewed the process itself positively, and believed that the resulting resources were user-friendly, valuable, and informative (Shriane *et al*. 2024). The present paper specifically focuses on the co-design process, examining how co-designers shaped discussions related to content needed for the physical activity component of the project in addition to presenting a qualitative analysis of the content and discussions that occurred within the co-design workshops. These collaborative efforts directly informed the physical activity resources developed as part of the broader project. Findings are presented as per the guidelines set out in the Standards for Reporting Qualitative Research (O’Brien *et al*. 2014).

Qualitative data were collected through a series of online workshops and analysed using Braun and Clarke’s reflexive thematic analysis (RTA) (Braun *et al*. 2022). The six-phases of RTA were employed, offering flexibility and a hybrid inductive-deductive approach: (i) familiarization; (ii) coding; (iii) initial theme generation; (iv) reviewing and developing themes; (v) refining, defining, and naming themes; and (vi) producing the report (Pacheco-Romero *et al*. 2021, Braun *et al*. 2022, Proudfoot and Proudfoot 2022, Naeem *et al*. 2023). This methodological approach was also selected for its recognition and acceptance of researcher subjectivity (i.e. lived experiences of co-designers), as described below (Braun *et al*. 2022). Data were gathered without the application of predefined theoretical models and were analysed using a hybrid inductive/deductive RTA. First, initial coding and theme development were primarily inductive, based on the contributions from co-designers. Second, deductive elements were incorporated to identify key features relevant to the practical aims of resource creation. Finally, the lived experiences of both the co-designers and researchers were integrated using the broader literature on shiftworker health and wellbeing. Although this paper focuses on physical activity, the analysis was conducted across sleep, nutrition, and physical activity data. As part of the deductive process, consistent foundational themes were intentionally developed to ensure coherence and integration across the suite of resources. Collectively, this process informed the creation of co-designed physical activity resources for YSW (Carter and Little 2007).

### Participants

Ethical approval for the project was granted by the Central Queensland University Human Research Ethics Committee (ref. no. 0000024460). Recruitment took place between September 2023 and January 2024, with eligible co-designers recruited via social media and professional networks (Table 1). Advertisements were posted to relevant social media groups and channels (Facebook, LinkedIn, Instagram) that focus on shiftwork or shiftwork industries. Additionally, recruitment materials were made available via professional networks including the LinkedIn profiles of members of the research team. Interested individuals followed a link that was provided as part of the advertisement materials, to access an information sheet describing what was involved in the study. If they wanted to participate, they provided informed consent through a dedicated webpage (Qualtrics, Provo, UT). Three series of workshops were undertaken (see below). The first group of participants recruited in this way participated in the first and second workshop series. A second group of participants was recruited in the same way for the third workshop series. A total of 42 co-designers were enrolled, with 36 (80%) attending at least one workshop (six co-designers withdrew after enrolling). Participants were compensated with a 150 AUD gift card per workshop in line with standard hourly rates for work of the majority of the participant population and recommendations from the Australian National Health and Medical Research Council (National Health and Medical Research Council 2019). The sample size (*n* = 36) was based on workshop feasibility (i.e. a maximum of 10 participants per workshop to ensure all voices and perspectives were appropriately captured) and the quality of data (i.e. data richness) (Braun and Clarke 2021). Full eligibility criteria and demographic details are provided in Table 1. Of note, several categories of shiftworker were recruited, including ‘YSW’, ‘experienced shiftworkers (ESW)’, and ‘PSW’. Experienced shiftworkers were defined as individuals with at least five years in a shiftwork role. In contrast, no minimum duration was required for YSW, as those at the lower end of the age range (e.g. ∼18 years of age) were unlikely to have extensive shiftwork experience. The perspectives of individuals who had recently commenced shiftwork were considered particularly valuable, as they represent a key target audience for the resources developed through this project. Experienced and PSW were included in the study to capture insights into effective strategies and reasons for leaving shiftwork, contributing to a broader understanding of the target population. In addition to the participants, co-design input was provided by members of the research team (*n* = 12).

**Table 1.** Participant eligibility criteria and co-designer demographic details.^a^

Participant category^d^	Eligibility criteria^e^	Participants (*n* = 36)	Age (mean ± SD)	Gender (*n*, % female)	Industry^#g^(*n*, %)	Years of experience^f^(mean ± SD)
Young shiftworkers	Aged 18–25 years; and, Currently employed in full-time shiftwork.	14 (38.9%)	23.9 ± 1.8	11 (78.6%)	Healthcare and social assistance (12, 85.7%) Construction (1, 7.1%) transport, postal and warehousing (1, 7.1%)	2.8 ± 2.0
Experienced Shiftworkers	Currently employed in full-time shiftwork for 5 or more years.	9 (25.0%)	31.7 ± 9.0	3 (33.3%)^b^	Healthcare and social assistance (6, 66.7%) Public administration and safety (3, 33.3%)	15.4 ± 10.20
Previous shiftworkers	Previously employed in full-time shiftwork for 5 or more years; and, Ceased shiftwork within the last 5 years.	3 (8.3%)	40.0 ± 10.2	2 (66.6%)	Accommodation and Food services (1, 33.3%) Administrative and support services (1, 33.3%) Healthcare and social assistance (1, 33.3%)	17.0 ± 10.0^c^
Workplace health and safety experts	Current or recent experience in providing advice to/oversight of shiftworker health and wellbeing.	7 (19.5%)	47.7 ± 8.3	3 (42.9%)	Mining (7, 100%) Education and training (5, 71.4%) Healthcare and social assistance (5, 71.4%) Construction (4, 57.1%) Transport, postal and warehousing (4, 57.1%) Agriculture, forestry and fishing (3, 42.9%) Electricity, gas, water and waste services (3, 42.9%) Manufacturing (3, 42.9%) Professional, scientific and technical services (3, 42.9%) Public safety and administration (3, 42.9%) Administrative and support services (2, 28.6%) Information media and telecommunications (2, 28.6%) Accommodation and food services (1, 14.3%) Financial and insurance services (1, 14.3%)	18.4 ± 9.3
Science communications specialists	Current or recent experience in communicating health and/or science information with young adults.	3 (8.3%)	32.0 ± 2.9	3 (100%)	—	9.0 ± 4.2

^a^In addition to the participants described in this table, 12 academic experts (members of the research team) contributed to the co-design process. ^b^*n* = 1 participant preferred not to disclose their gender. ^c^Denotes years of experience as a shiftworker (i.e. prior to leaving shiftwork). ^d^Where necessary, participants self-selected the category they felt most qualified to represent (e.g. having both shiftwork and science communications experience; self-assessment for relevancy and recency of work). ^e^All participants confirmed that they were in Australia and have access to an internet-connected device to complete online questionnaires and participant in online workshops. ^f^‘Years of experience’ refers to years spent in a shiftwork role (for shiftwork participant groups) or years spent in current profession (for work health and safety experts and science communication specialists). ^g^Industry refers to the industry that a participant is currently employed within as per their designated participant category. Workplace health and safety experts were able to select more than one industry. Science communications specialists were not asked about industry given the specificity of their job role.

### Workshop facilitation and transcription

**Figure 1.**
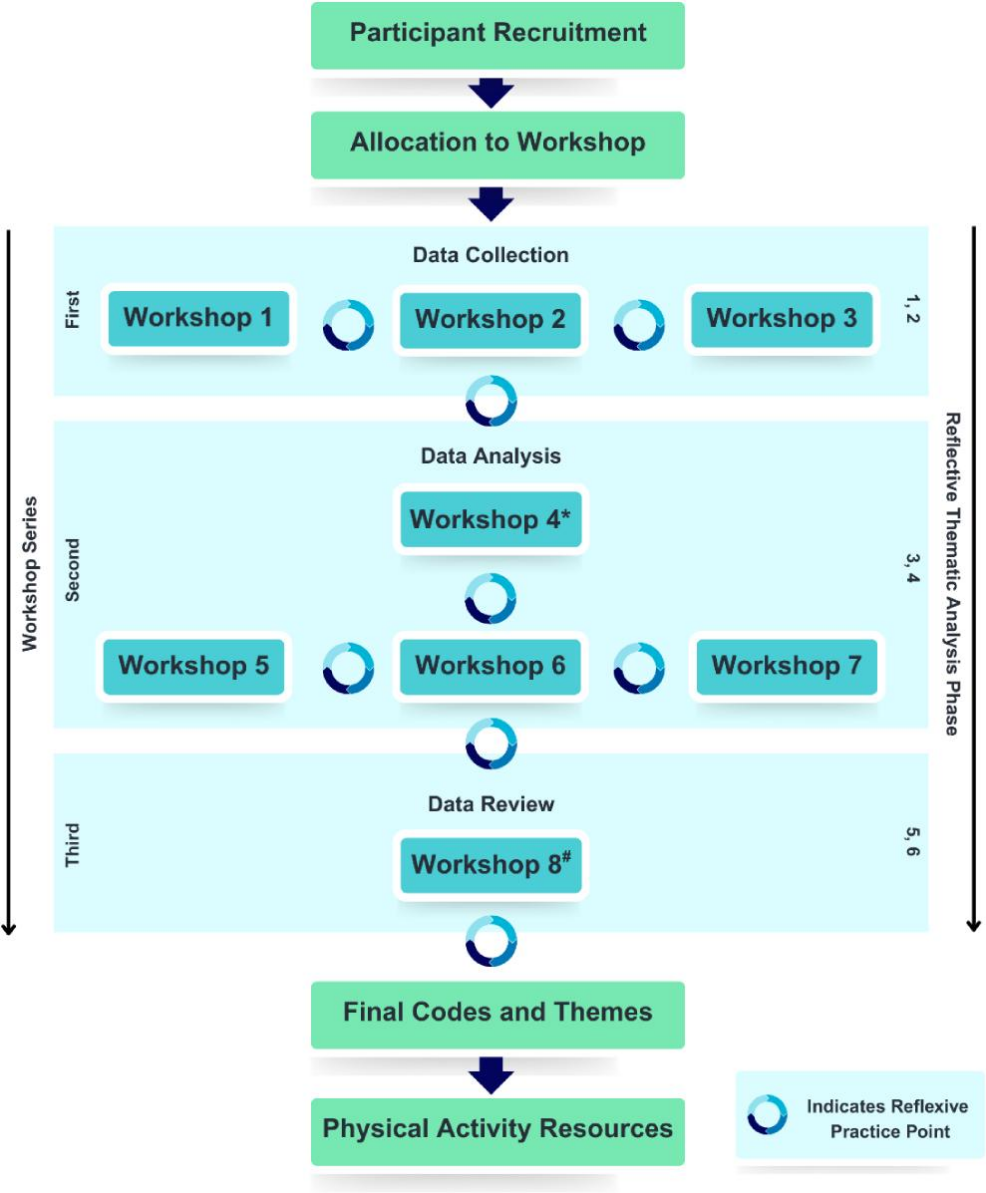
Workshop series. ^*^Denotes academic expert (project team) workshop, not involving lived experience co-designers. ^#^Denotes new young shiftworkers recruited for a stand-alone workshop, not involved in previous workshops. Figure adapted from our previous work (Shriane *et al*. 2025).

The study included a series of online workshops conducted via Zoom (Version 6.1.11), each lasting approximately 60 min (Fig. 1). The workshops were led by A.E.S. (project co-lead with expertise in sleep optimization for shiftworkers), alongside co-facilitators G.E.V. (project co-lead, senior sleep and physical activity researcher), and C.C.G., (shiftwork and chrononutrition expert). Each session was audio- and video-recorded, and meeting chat captured for transcription. Workshop sessions were automatically transcribed using the Zoom transcription feature, with the resulting files exported to Microsoft Word. A.E.S. manually transcribed each workshop from the recording using a linear denaturalized method (Azevedo *et al*. 2017), anonymizing participants with acronyms based on their identified participant category and unique identifiers assigned at enrolment, as per Table 1 (e.g. YSW1, WHS3). The manually-developed transcriptions were then cross-checked against the automatically-created versions for correctness and completeness.

After each workshop, the facilitators (A.E.S., G.E.V., C.C.G.) held debrief sessions to review outcomes, challenges, key learnings, and implications for data analysis. These sessions were recorded (see Reflexive Practice Points in Fig. 1), with summary notes created by A.E.S. to reflect debrief discussions. No personal information was included, and co-facilitators (G.E.V., C.C.G.) reviewed the transcriptions for accuracy. The transcriptions informed later project activities, and workshop guides remained consistent within each series.

### Data collection: first series

The first series of workshops (Workshops 1–3) aimed to address the research question by inductively exploring co-designer recommendations on physical activity topics for YSW. The number of participants from each category [e.g. YSW, ESW, WHS experts, science SC,AE] differed slightly between workshops, but the total number of participants was either 10 (Workshop 1 and 3) or 9 (Workshop 2; total number of participants in first series = 29) [Participant breakdowns: W1 (YSW = 2; ESW = 2; PSW = 1; WHS = 4; SC = 1); W2 (YSW = 2; ESW = 4; WHS = 2; SC = 1); W3 (YSW = 4; ESW = 3; PSW = 1; WHS = 1; SC = 1); W4 (AE = 12); W5 (ESW = 2; WHS = 4; SC = 1); W6 (YSW = 1; ESW = 4; PSW = 2; WHS = 2; SC = 1); W7 (YSW = 5; ESW = 3; PSW = 1); W8 (YSW = 7).]. Workshops were conducted in line with the workshop guide (see Table 2) developed by A.E.S., G.E.V., and C.C.G., pilot tested with the broader project team (S.A.F., G.R., T.K.-A., M.S., R.S., M.J.W.T., J.L.P.), and revised based on feedback from the team. The workshop guide consisted solely of open-ended questions, with follow-up when required. This process was undertaken in line with the framework for developing a semi-structured interview/workshop guide described by Kallio *et al*. (2016).

**Table 2.** First series workshop discussion guide.

Topic	Primary guiding question	Additional discussion points
Physical activity	What do we need to tell young shiftworkers about physical activity?	What do young shiftworkers already know about physical activity? How would this information differ from the general population? How would this information differ from an older/more experienced shiftworker? What information is general, and what needs to be tailored to certain industries?
Communication	How do we communicate this information with young shiftworkers?	What resources do young shiftworkers want or need? Where are young shiftworkers getting their health and wellbeing information? What should we avoid when communicating this information?

### Data analysis: development of codes and themes

Following transcription, data cleaning was conducted in Microsoft Word thereafter, which involved colour-coding keywords based on their semantic content, as outlined in the second stage of RTA (Naeem *et al*. 2023). In keeping with inductive approaches (Fereday and Muir-Cochrane 2006, Vears and Gillam 2022), pre-existing theoretical frameworks or models were not applied at this stage.

In alignment with the third step of RTA (Naeem *et al*. 2023), a ‘cut and categorize’ approach was utilized after colour-coding of keywords (Basit 2003). Specific sections of text (such as phrases or words) were extracted from the colour-coded material if they were directly related to shiftworkers’ physical activity. These extracted ‘codes’ were then grouped based on shared semantic meaning, thus forming broader themes, as outlined in the fourth stage of RTA (Naeem *et al*. 2023). Reflective debriefing was used, rather than inter-coding reliability of themes to align with our constructivist approach, which prioritized shared understanding, contextual meaning, and reflexivity (Braun and Clarke 2021). Following the development of codes and themes by A.E.S., G.E.V., and C.C.G. reviewed for completeness. Theme names were then developed and assigned, based on their ability to accurately and succinctly capture the themes’ content and communicate their meaning. As workshops included guiding questions related to sleep and nutrition in addition to physical activity, the analysis process was undertaken simultaneously across the domains. As a result, codes and overarching themes were developed that incorporated multiple health behaviours. Analyses and content related to sleep and nutrition have been published elsewhere (see above).

### Data analysis: second series

The second series of workshops included four sessions. Workshop 4 involved AE. Workshops 5 to 7 were attended by the same co-designers who participated in the first series (Workshops 1–3), with each invited to one of the three scheduled sessions (two co-designers did not return). During this second series, the codes and themes developed from the initial data were presented, first to the AE (Workshop 4), and then to the returning co-designers (Workshops 5–7). The co-designers were presented with this information in the context of the resources (i.e. they were given examples of how these codes and themes would be translated within the resulting resources). These workshops followed the same approach and guide as the first series (see Table 2 and Supplementary Material in Shriane *et al*. (2025)). Transcription and analysis were also consistent with the first series, with adjustments made to the codes and themes before the third series (Workshop 8).

### Data review: third series

In the third series (Workshop 8), the developed codes and themes were presented to a new group of YSW (i.e. who had not yet been involved in the project), using the same approach as previous workshops for shiftworkers and the same workshop guide as in the second series (see Table 2 and Supplementary Material in Shriane *et al*. (2025)). The goal was to review the data and further refine the codes and themes, ensuring that the themes and resource content identified via the first and second series also reflected the perspectives of a new group of co-designers. Afterward, the transcription process from earlier series was repeated, finalizing the data for resource creation.

### Procedure: conceptual model development and resource creation

Following the transcription of the third series of workshops, the research team generated codes and themes based on the data using RTA. These were then compared against contemporary evidence-based advice on physical activity for shiftworkers, sourced from peer-reviewed literature and reputable public health resources, to examine points of alignment and divergence. This step, consistent with the initial stages of conceptual model development as outlined in RTA (Naeem *et al*. 2023), informed the creation of a practical framework to guide resource development. Practical considerations such as feasibility, cost, and time constraints were also integrated into this process.

### Positionality and reflexivity

Given the collective personal and professional experience of the project team, positionality was a vital consideration in this project. One project lead (A.E.S.) has extensive experience as a shiftworker, and many members of the project team have a lived experience of shiftwork. Collectively, this experience provided a broad and diverse understanding of the physical activity challenges faced by YSW, in addition to significant subject matter expertise, and sound knowledge of how to investigate these topics and interpret findings (Holmes 2020). Given the lived experience of the project team, a co-design methodology was employed to ensure the voices and perspectives of shiftworkers and relevant experts directly informed the content and delivery style of the resulting resources (Kitagawa and Kitagawa 2023). The expertise and experiences of the research team also played a role in ensuring the content produced was evidence-based and informed by best practice science. The team comprised researchers with varied expertise, including but not limited to shiftwork, physical activity, and sleep, which enabled interdisciplinary input and ongoing critical reflection throughout the process. Rather than aiming to minimize bias, the research adopted a reflexive approach that acknowledged this positionality and the ways in which backgrounds, assumptions, and experiences shaped the research process and interpretation of findings. This openness to reflexivity was central to the co-design process (see below). Further information on the research team’s considering of positionality (Cunliffe *et al*. 2013) is outlined in the Supplementary Material of Shriane *et al*. (2025).

Reflexive Statement: Reflexivity played a vital role in this project due to the research team’s professional and personal backgrounds. The team recognized that these experiences could shape the RTA process, as it naturally involves researcher perspectives influencing the analysis of data. To address this, the project incorporated iterative feedback loops, promoting regular team discussions and continuous collaboration with co-designers, as illustrated in Fig. 1. This approach ensured that data interpretation remained both transparent and robust, minimizing the potential for biases to unduly affect the project. Furthermore, to maintain awareness of these biases and the strategies employed to address them, the team conducted regular debriefing sessions, encouraging reflection on key decisions, processes, and responses throughout the project.

## RESULTS

**Figure 2.**
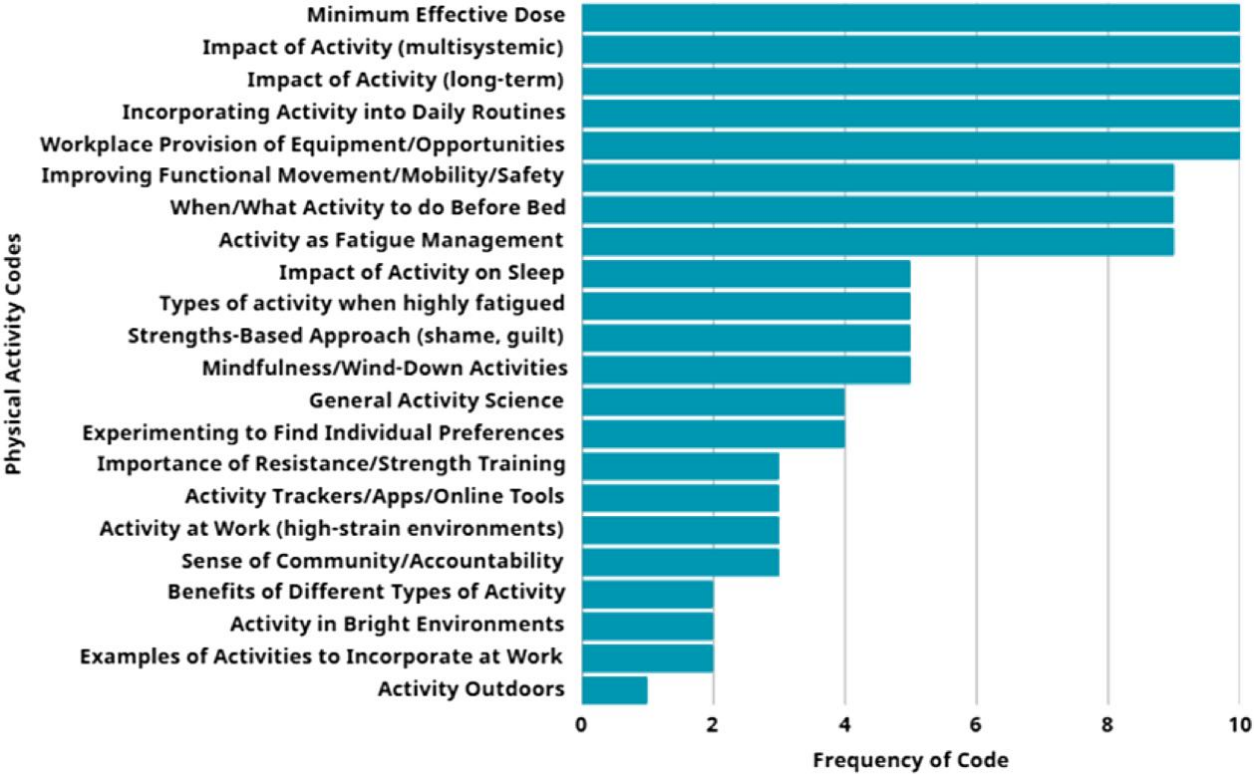
Frequency of codes articulated by co-designers.

**Figure 3.**
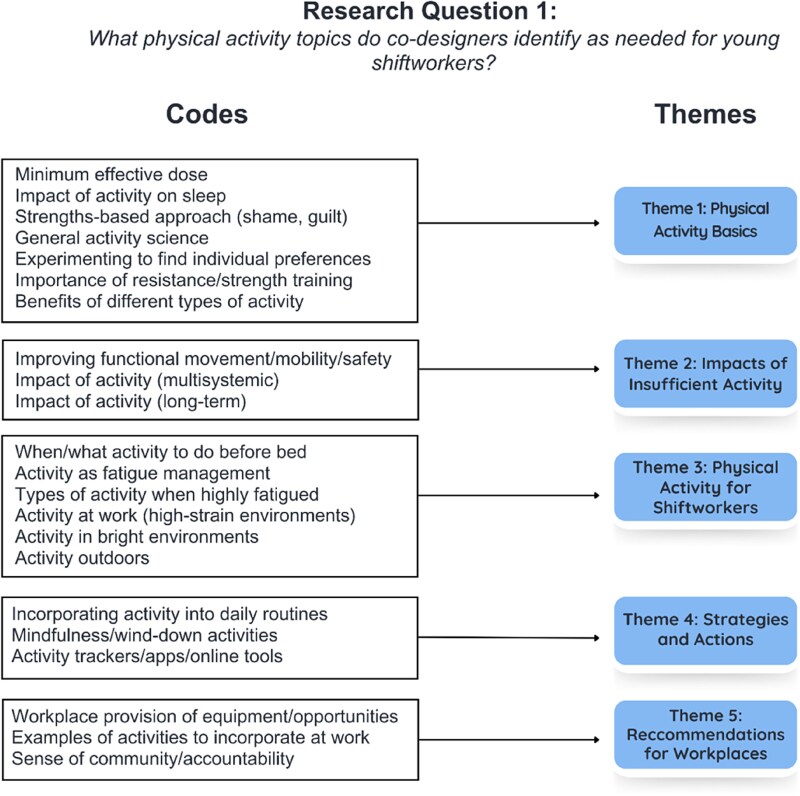
Physical activity resource codes grouped by theme.

Analysis of transcripts from the first series of co-design workshops produced 183 initial unique codes, 22 of which were associated with physical activity (the rest related to sleep or nutrition—reported elsewhere). The frequency of each code occurring during co-design workshops is displayed in Fig. 2. These frequencies are presented to support a clearer understanding of how commonly certain topics were raised and to help guide the scope and emphasis of the resulting resources. The 22 codes were categorized into five themes: physical activity basics, impacts of insufficient physical activity, physical activity for shiftworkers, strategies and actions, and recommendations for workplaces (see Fig. 3). When the themes were presented to existing co-designers in the second series of workshops, and then again to the newly recruited co-designers in the third series of workshops, no changes were made to overarching themes, though additional information was provided that was used to populate the content of the resulting resources. Identification codes reflecting participant categories have been included where relevant. These codes include: YSW, ESW, PSW, WHS, and SC specialists.

### Theme 1: physical activity basics

The need for improved education on physical activity basics for YSW was revealed during co-design workshops. The group reported that knowledge of general physical activity science could empower YSW to engage in achievable, lifestyle-compatible exercise. One workplace health and safety expert shared:

If we’re really pitching this as a sort of entry-level or information that’s covering all of the basics, then it really needs to look what the research says about minimum effective dose…I think people might be relieved to know that the minimum effective dose is still achievable for most people. (WHS 3)

The concept of a minimum required dose of physical activity needed to see benefits was among the most frequently highlighted topics. Co-designers felt this could motivate YSW, who often feel shame or guilt around being unable to undertake long, high-intensity workouts regularly. One experienced shiftworker revealed:

People will generally fall back on no physical activity if they feel shamed or guilted. Any type of movement is better than no movement. (ESW 7)

The topic of shame and guilt around physical activity was brought up by other participants, with many noting that these types of negative beliefs hinder their capacity or willingness to engage in physical activity.

If you’re someone that exercises regularly, or exercises every day, and every time you have a block of night shifts you stop exercising, you’re out of routine and you feel lazy. (ESW 2)

Participants highlighted the need to take a strengths-based approach to designing resources for YSW.

While structure is good, I don’t think you should beat yourself up if you can’t go and exercise every single day. What suits you sometimes may not suit you at other times. (WHS 1)

The beneficial impact of physical activity on sleep was another important aspect of education emphasized strongly among co-design participants. As one young shiftworker said:

It’s also about the relationship between [physical] activity and sleep, and that being active will help you to sleep. (YSW 4)

Co-designers also felt that education regarding the benefits of different types of activity could be another valuable aspect of education for YSW. An experienced shiftworker explained:

…is it a gentle sort of work out that might elevate your heart rate as opposed to a high intensity, interval training type of exercise, which is probably not ideal before doing nightshift, because it might deplete a lot of your energy before work. So, just looking at the type of training through the lens of what is most appropriate. (ESW 1)

The importance, and potential functional benefits, of resistance training was also spoken about in this context by co-designers. One science communications expert highlighted:

Because a lot of shiftwork can be quite physical, you’re on your feet all day, it’s a good reminder that exercise can be about improving that function and supporting muscles through resistance training. (SC 2)

In addition to educating YSW on general physical activity science concepts, co-designers also highlighted the importance of YSW having the opportunity to experiment and determine what works best for them. Co-designers noted that if physical activity habits can align with individual preferences, physical activity is more likely to be sustainable. One workplace health and safety expert shared:

*…*what that actually looks like depends on the individual and what they enjoy and what they’re willing to do… just keeping it simple in relation to getting people moving, whether it’s high intensity, low intensity, resistance-based, cardio-based, I think it’s about just getting people moving on a regular basis. (WHS 5)

### Theme 2: impacts of insufficient physical activity

The cumulative impacts of insufficient physical activity were spoken about by co-designers at length during workshops. Participants placed particular emphasis on physical activity promoting shiftworkers’ ability to continue their jobs comfortably over the long-term. As one young shiftworker noted:

When you get a bit older, you realise that if you don’t use the opportunity to move your body and strengthen your body, then unfortunately, you do get to a point in your life where if you don’t use it, you lose it. You won’t be able to move your body in certain ways, you’ll feel uncomfortable, you’ll have pain. (YSW 3)

One young shiftworker spoke about experiencing the benefits of increasing physical activity after previously having struggled to find the time, saying:

Early in my career, when I wasn’t really exercising and I was very new to shiftwork, I just didn’t find that I had time to exercise…But then, as I felt like my health and my energy sort of declined, integrating exercise did absolute wonders for me… I know that’s only anecdotal, but I felt like I was achieving a lot more, in a mental, physical and emotional sense. (YSW 5)

This prompted the group to discuss the beneficial impact of physical activity on functional mobility and safety, which was one of the most strongly emphasized topics throughout co-design workshops. One science communications expert noted:

‘Exercise can be about improving that function and supporting muscles through resistance training–it can be about helping to reduce injuries or supporting being on your feet all day not causing pain’ (SC 2)

An experienced shiftworker added the importance of flexibility in this regard to the discussion:

Because shiftworkers often are sitting a lot, standing a lot, or are up and down, maintaining that flexibility will always be important for reducing the chances of injury. (ESW6)

### Theme 3: physical activity for shiftworkers

The need to find the most effective ways of incorporating physical activity into shiftworkers’ busy, unique schedules was discussed at length by co-design participants. Timing of different types of physical activity, especially in relation to bedtime were some of the most frequently discussed considerations among co-design workshop participants. A workplace health and safety expert described:

Timing of exercise and type of exercise also needs to be considered–e.g. boxing before bed may stimulate and keep people awake whereas a walk or cycling may not. (WHS 5)

An experienced shiftworker also raised the importance of education for shiftworkers regarding how to time different types of physical activity, saying:

Often times though, physical activity just before you’re trying to fall asleep may help you to feel physically tired, but you’ll be buzzing from the exercise, so again, you could be shooting yourself in the foot. (ESW6)

Co-designers strongly emphasized their experience of physical activity helping to manage fatigue and also acknowledged the benefit of bright environments to facilitate these benefits. One young shiftworker explained:

*…* if I don’t exercise, I feel worse going into my next shift, and I feel more fatigued. Whereas, if I go for a walk for like 30 minutes before my shift starts, I feel better and I have more energy. (YSW 10)

Participants acknowledged, however, that shiftworkers would benefit from guidance on what types of activity to perform when feeling highly fatigued. As an experienced shiftworker said:

*…*in terms of exercising when fatigued, do you have a higher risk of injury, so therefore you may need to choose different exercises because you’re fatigued. (ESW8)

It was also noted by co-designers that some work environments are more physically demanding than others, and that shiftworkers in these roles may not need to perform as much physical activity outside of work. One workplace health and safety expert shared:

Some people will have a very heavy, physical workload while they’re at work… you might also get them to consider that, so they don’t feel guilty just because it’s recommended that they should do a certain amount of exercise. They need to consider what they’ve done in their job. (WHS7)

### Theme 4: strategies and actions to improve physical activity for shiftworkers

Throughout the co-design workshops, participants discussed strategies and actions that had effectively supported their own physical activity. They also shared additional suggestions, originating from peer discussions within the cohort, that they had not implemented themselves. The most frequently highlighted strategy was incorporating physical activity into normal daily routines to make it both achievable and sustainable for YSW. One experienced shiftworker discussed their experience, revealing

For me, a lot of it comes down to normal routine. If you’re someone that exercises regularly, or exercises every day, and every time you have a block of night shifts you stop exercising, you’re out of routine and you feel lazy. So, for me, it’s important not to alter that routine. (ESW2)

Numerous co-designers also emphasized the value in incorporating mindfulness and/or low-impact wind-down activities, which can be highly beneficial and take less time and effort than completing an intense workout after a long shift. A young shiftworker shared:

I think people need to consider mindfulness as well. I think it’s important to take care of your mind as well as your body and incorporating mindfulness or meditation or yoga to help ease the stress of your job, and the long and tiring impacts of shiftwork. (YSW7)

Utilizing mindfulness and/or low impact wind-down activities was emphasized not just as a way to reduce the stress impacts of shiftwork, but also to facilitate improved sleep. One young shiftworker said:

If you’ve come home from a day shift, here’s some things that take 15 minutes, even if they’re things that tie into winding down before sleep. (YSW12)

An ex-shiftworker echoed a similar sentiment, explaining that yoga has helped them in the past to wind down before sleep:

One of the things that I’ve found to be really beneficial is yoga—you don’t have to go for a 5 km run post-night-shift, but instead, you can prioritise that ability to unwind leading into sleep. (ESW2)

Leveraging apps, online tools and/or physical activity trackers was another strategy to improve physical activity that was raised by numerous co-designers. One experienced shiftworker suggesting:

There’s a lot of apps that are available on your phone that can be customised to the type of exercise that you want to do, so people should use those if they’re helpful. (ESW4)

Participants also highlighted the potential for technology to ‘track’ the amount of time workers spend sitting, and to prompt them to be physically active. One experienced shiftworker suggested:

If you’re working at a desk all the time, there could be something that flashes up recommending that you move. (ESW9)

### Theme 5: recommendations for workplaces

Co-designers’ recommendations for workplaces wanting to support YSW to be physically active focused mostly on workplace provision of opportunities and equipment. Some examples of activities to incorporate at work to reduce the time burden of physical activity for shiftworkers were also discussed in this context. One workplace health and safety expert shared:

*…*looking at what organisations can do to support them–whether it’s equipment or fitness programs or walking from office to office, or just providing something to encourage them, and getting supervisors out there and creating that team environment that encourages exercise or physical activity, certainly within their rostered time as well. (WHS5)

In addition to reducing the time burden associated with physical activity, workplace provision of equipment was raised as a way to increase accessibility for YSW wanting to be physically active. One young shiftworker explained:

I think accessibility is another important factor as well…if we have a gym or just a treadmill or some kind of small exercise station, that would be really helpful. (YSW2)

Employers incentivising physical activity was discussed by numerous co-designers as a strategy for workplaces wanting to support YSW to be physically active. One experienced shiftworker suggested using incentives to reward employees for meeting yearly fitness assessment goals:

*…*maybe incentivising something around yearly fitness assessments…whether it’s gym work or cardio, or something during rostered time. That’s something that workplaces could look at. (ESW2)

Participants also spoke about the sense of community and accountability created as a result of physical activity at work, one ex-shiftworker sharing how this was beneficial in a previous role for them:

We participate in what they call a [funded health and wellbeing program that provides gym access], and we’ve found it really good. It has actually created a sense of community amongst the different officers when they’re attending the local swimming pool or the local gym. (ESW2)

## DISCUSSION

Using co-design principles, the present study aimed to identify content that should be incorporated within a suite of resources to support physical activity in YSW. This study sits within a larger participatory co-design project that aimed to develop resources across the physical activity, sleep, and nutrition domains. Five themes were identified, ranging from individual-level science information (physical activity basics, impacts of insufficient physical activity) to practical strategies (physical activity for shiftworkers, strategies and actions to improve physical activity) and recommendations for workplaces. Within these themes, a range of recommendations and potential strategies were identified relating to how relevant information could be communicated with YSW to maximize uptake. The findings of this study have been used to develop a suite of resources that is freely available for YSW worldwide (www.healthyshiftwork.com). The five physical activity themes identified in this study were used to directly inform the content of the resulting resources, with infographics and a dedicated page on the website created for each theme.

Across many of the identified themes, the need to provide motivational and encouraging messaging within information provided for YSW was consistent. This ranged from the perceived need to ensure YSW are aware of the minimum effective dose for physical activity, to practical and personal benefits. Broadly, co-designers believed that it was important for information related to physical activity to be presented in a positive light, avoiding fear- or shame-based communication strategies. Additionally, it was considered important to acknowledge and leverage the unique opportunities available to shiftworkers (e.g. access to gyms during off-peak hours, having more free time during daylight hours). This strategy, often referred to as a strengths-based approach, is considered by some to be one of the more effective strategies for changing individual behaviour related to physical activity (Warburton and Bredin 2019). That is, rather than approaching behaviour change via a negative, fear-based, or shame-based lens, it is generally considered more effective to leverage existing strengths and capabilities (Zelle *et al*. 2016, Hamer *et al*. 2021). A strengths based approach may also be more likely to increase engagement and longer-term uptake (Stover *et al*. 2024), particularly when coupled with advice that is specific to an individual’s own circumstances (i.e. as a shiftworker) (Patrick *et al*. 2013). Conversely, it may be more difficult to engage with educational approaches that focus on negative outcomes (Warburton and Bredin 2019). Avoiding potential barriers to engagement is especially important in this context, as the success of the online and multimedia resources developed in this study depends on active participation from YSW. This contrasts with more traditional approaches, such as in-person, clinical, or workplace-based interventions, which may rely less on individual initiative.

Co-designers generally recommended physical activity content that aligns with the current evidence base. For example, co-designers believed physical activity was important due to both health and functional outcomes (Proper *et al*. 2002, Yang *et al*. 2010, Kokkinos 2012) and encouraged the consideration of factors such as the type (Brellenthin *et al*. 2022), timing (Mancilla *et al*. 2020, Willis *et al*. 2020), and duration (Powell *et al*. 2011) of physical activity. Moreover, many recommendations related to strategies that may be specifically appropriate for shiftworkers (e.g. use of pre-shift physical activity as an alertness management strategy) were supported by evidence (Easton *et al*. 2024). However, it is worth considering that many of the co-designers, while having lived experience with shiftwork or other relevant areas (e.g. science communication) were not experts in the physical activity. As such, there were some recommendations that were approached with caution when developing the resulting resources. For example, several co-designers discussed the potential impact of physical activity related to occupational tasks. While the co-designers rightfully discussed that undertaking physical activity at work may be relevant when planning non-work physical activity, it is worth noting that the health benefits of physical activity during work time (i.e. occupational physical activity) are somewhat limited (Holtermann *et al*. 2012, 2017). At work, low intensity, repetitive physical activity tends to be undertaken over extended periods, with limited opportunities for self-pacing during the activity, increased stress, and potentially restricted subsequent recovery periods (Janssen and Voelcker-Rehage 2023). As a result, occupational physical activity is paradoxically associated with poorer health outcomes (e.g. cardiovascular disease risk)—and this is known as the physical activity paradox (Temporelli 2021). Moreover, physical activity undertaken at work can also result in a decrease in non-work physical activity, further compounding this problem (Kolbe-Alexander *et al*. 2019). As such, we must be cautious about extolling the virtues of occupational physical activity and instead consider the impact of work-based physical activity on leisure time physical activity. Recommendations such as this (i.e. those that do not align with the evidence-base) were not reflected in the final resources. All content was reviewed by the research team as part of the co-design process to ensure consistency with current best practice guidelines and scientific evidence.

**Figure 4.**
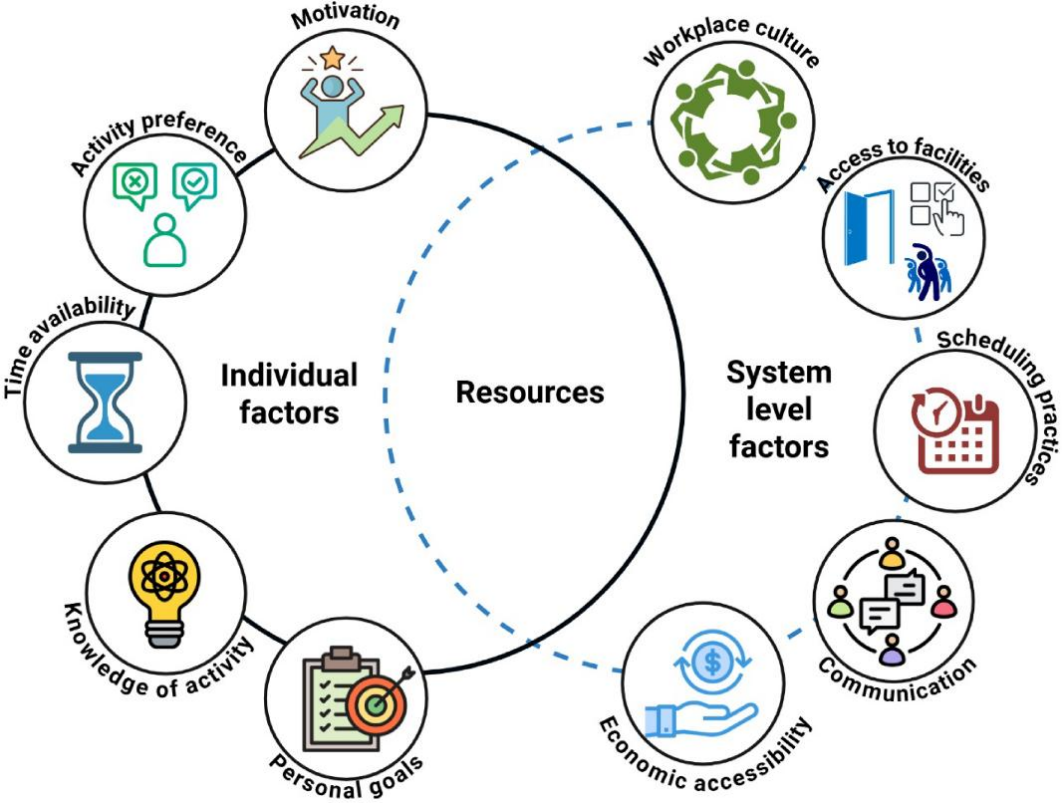
Individual and system level factors supporting physical activity for shiftworkers.

The importance of physical activity being integrated into and supported by the work environment was also a theme identified by the co-designers. Critically, this theme related to the importance of considering not just the individual-level factors that can support physical activity, but also system-level factors. For many shiftworkers, it is these work-related system factors beyond simple hours of work (e.g. broader factors such as organizational culture, support, and resources) that directly impact their capacity to consistently undertake physical activity (Pronk 2021, Safi *et al*. 2022). The findings of the present study highlight the importance of this interaction between system and individual factors (see Fig. 4). For the resources developed as part of this project to be as impactful as possible, it appears that application at the workplace or industry level may be needed. Physical activity interventions that are undertaken via workplaces are more likely to be successful, particularly over the long term, than many focused at the individual level alone (e.g. primary care interventions) (Murray *et al*. 2017). Ideally, a whole-system approach would be taken, whereby workplaces would incorporate strategies designed to support physical activity (e.g. on-site exercise facilities, providing access to physical activity smartphone applications or challenges, providing consistency with rostering to allow shiftworkers to engage in organized sports or exercise classes, providing healthcare support where appropriate), in addition to the critical individual-level focus (e.g. motivation, practical strategies). Economic accessibility may also influence what strategies are feasible, and in some cases, support from local councils or government initiatives (e.g. cycle-to-work schemes) could help enable implementation. Indeed, this whole of systems approach to promoting physical activity has further implications for actual ‘job design’ considerations, ensuring that opportunities for increasing physical activity are not only seen as separate to work itself, but integrated into more health task design (Naweed *et al*. 2022).

### Strengths and limitations

There are both strengths and limitations that should be considered when interpreting findings. A key strength of the present study is the use of a systematic co-design approach, which provides a framework for other researchers and practitioners who wish to employ co-design methodologies for health interventions and/or with young people. The comprehensive nature of the approach also means we can be confident that the findings, and resources themselves, accurately reflect the needs and perspectives of YSW. However, future research should be undertaken to determine whether the co-designed resources have a meaningful impact on physical activity in YSW. It is possible that while co-designers provided information that aligned with their preferences and circumstances, this information may not result in actual behaviour change for the co-designers or other shiftworkers. Future research will ideally be undertaken to evaluate the direct impact of the resources on the long-term health of YSW. Additionally, to preserve anonymity and confidentiality, demographic information of co-designers collected did not include income, education level, or shift types undertaken. This type of information may impact health behaviours, and as such would ideally be collected in future studies, including those where health impacts are evaluated. Additionally, the population involved in the present study were largely based in Australia. While this means our findings are reflective of the Australian perspective, it is possible that international generalisability is limited. As part of an evaluation process, differences in effectiveness between populations would ideally be examined. The potential impact of researcher positionality and reflexivity must also be noted. Given the expertise and associated positionality of the research team, we found it was critical to engage in a reflexive process throughout the data collection and analysis process. We recommend incorporating reflexivity as a core practice in future co-design projects.

## CONCLUSION

Physical activity is critical for health and is a significant challenge facing YSW in Australia and beyond. The co-design approach used in the present study has established a clear scope for the content necessary to support this population in achieving sufficient physical activity. Based on the target content, a suite of freely-available resources have been developed, though future research is needed to evaluate their uptake and effectiveness. It is likely that both individual- and system-level content and application will be needed to ensure their effectiveness.

## Data Availability

De-identified data that supports the outcomes in this manuscript is available upon request from the corresponding author.
